# Climate-informed surveillance for climate-sensitive infectious diseases in Somalia: early warning systems, policy gaps, and preparedness priorities

**DOI:** 10.3389/fepid.2026.1907542

**Published:** 2026-09-11

**Authors:** Abdikadir Ahmed Hassan, Liibaan Abdulahi Sudi, Mohamed Daud Mohamed, Iqro Omar Ibrahim, Yakub Burhan Abdullahi

**Affiliations:** 1 Faculty of Health Sciences and Tropical Medicine, Somali National University, Mogadishu, Somalia; 2 Faculty of Health Sciences, Salaam University, Mogadishu, Somalia; 3 Center for Health Research and Innovation, Somali National University, Mogadishu, Somalia; 4 Mogadishu Institute of Health, Mogadishu, Somalia

**Keywords:** anticipatory action, climate-sensitive infectious diseases, early warning systems, fragile health systems, one health surveillance, Somalia

## Abstract

Climate variability is reshaping infectious disease risk through ecological, environmental, social, and health-system pathways. However, its operational implications remain insufficiently defined in fragile and conflict-affected settings. Somalia is a critical case because recurrent droughts and floods intersect with displacement, food insecurity, weak water and sanitation infrastructure, disrupted immunisation, livestock dependence, and constrained health-system capacity. This narrative review synthesises evidence on climate-sensitive infectious disease risks in Somalia, examines existing surveillance and early warning systems, identifies policy and implementation gaps, and proposes priorities for a climate-informed surveillance-to-action model for infectious disease control. The review distinguishes directly climate-sensitive infections, including cholera, malaria, dengue, and Rift Valley fever, from indirectly climate-amplified vaccine-preventable diseases, including measles, diphtheria, and polio. While Somalia has important surveillance and early warning assets, including EWARN, IDSRS/DHIS2, FEWS NET, FSNAU intelligence, WASH monitoring, livestock signals, and community-based reporting, these streams remain insufficiently integrated into district-level risk assessment and pre-financed preparedness action. Therefore, the central challenge is not the absence of warning signals but the failure to convert them into timely, disease-specific, and accountable responses. We propose a surveillance-to-action framework based on multisource signal detection, integrated district risk assessment, disease-specific trigger decisions, early preparedness actions, and feedback, accountability, and learning. Closing Somalia's warning-to-action gap requires pre-agreed disease-specific triggers, One Health coordination, flexible anticipatory financing, strengthened district surveillance capacity, and equity-centred outreach to internally displaced persons, pastoralist and nomadic communities, zero-dose children, malnourished populations, and hard-to-reach areas. Climate-informed surveillance should be understood not merely as better data collection but as an anticipatory public health function designed to act before climate shocks become infectious disease emergencies.

## Introduction

Climate variability modifies the risk of infectious diseases through ecological, environmental, social, and health system pathways. Rainfall anomalies, droughts, flooding, and temperature shifts can alter vector habitats, disrupt water and sanitation access, drive food insecurity, and precipitate population displacement, each of which amplifies the transmission potential of multiple pathogens (1, 2). Evidence from sub-Saharan Africa documents important associations between temperature variability and morbidity and mortality, although the available evidence remains heterogeneous and methodologically limited (3). Critically, climate acts as a risk multiplier operating through social and institutional pathways, not as an independent determinant of outbreak occurrence (4).

Somalia is a critical case study. Recurrent droughts and floods, protracted conflict, large-scale displacement, acute food insecurity, and a health system operating with severe health workforce constraints and low, uneven routine immunisation coverage create compounding and intersecting vulnerabilities (5, 6). Regional surveillance coordination and information sharing are also important complements to national preparedness (7).

Two analytically distinct disease categories require different approaches to preparedness. Directly climate-sensitive infections, including cholera, malaria, dengue, and Rift Valley fever, are influenced by climatic and environmental conditions through water, vector, and ecological pathways (1, 2, 8, 9). Indirectly climate-amplified vaccine-preventable diseases — measles, diphtheria, and polio — are primarily driven by displacement, malnutrition, disrupted immunisation, and reduced healthcare access, all of which are intensified by climate shocks (10, 11). Preserving this distinction is essential for disease-specific surveillance and response design.

Somalia's deeper challenge is not merely delayed outbreak detection. Climate forecasts, water, sanitation, and hygiene (WASH) indicators, displacement data, nutrition metrics, livestock signals, and community-level intelligence are generated by multiple actors but remain insufficiently integrated into district-level risk assessment, the Integrated Disease Surveillance and Response System (IDSRS), One Health coordination, or pre-financed response triggers, creating a warning-to-action gap (12–14).

Previous Somalia-focused reviews have examined climate-related infectious disease risks (5) and the development of One Health in the country (14). This critical narrative review extends the literature by distinguishing climate-sensitive infections from vaccine-preventable diseases indirectly amplified by climate shocks and by shifting attention from climate–disease associations to the warning-to-action gap. It synthesises evidence on disease risks and existing early warning systems, identifies governance and implementation barriers, and proposes a district-level surveillance-to-action framework linking multisource signals with institutional authority, anticipatory financing, One Health coordination, equity targeting and accountability.

## Review approach

### Search strategy and information sources

We conducted a critical narrative review to examine climate-sensitive infectious disease risks in Somalia, assess existing surveillance and early warning systems, identify policy and implementation gaps, and develop contextually relevant preparedness priorities. Peer-reviewed and authoritative institutional sources published between January 2000 and June 2026 were considered, with searches updated in June 2026. PubMed/MEDLINE served as the principal bibliographic database for identifying peer-reviewed evidence, whereas Google Scholar was used as a supplementary source to identify additional relevant literature. Targeted searches were also conducted across authoritative institutional sources, including the World Health Organization, WHO Regional Office for the Eastern Mediterranean, Integrated Food Security Phase Classification, Famine Early Warning Systems Network, Food Security and Nutrition Analysis Unit, and Somalia Health Cluster. Search concepts combined Somalia with terms relating to climate change and variability, drought, flooding, rainfall, infectious and vaccine-preventable diseases, surveillance, early warning, EWARN, IDSR/IDSRS, DHIS2, One Health, water, sanitation, hygiene, displacement, nutrition, food insecurity, community surveillance, anticipatory action, and fragile health systems. The searches were iterative and concept-based, consistent with the analytical purpose of a critical narrative review.

## Evidence selection and contextualisation

A Somalia-first approach guided the selection of evidence. Priority was given to Somalia-specific epidemiological studies, outbreak investigations, surveillance evaluations, relevant reviews, and authoritative operational and policy documents. Regional and global evidence was selectively included when Somalia-specific evidence was limited or when it provided a directly relevant methodological, conceptual, or operational lesson. Evidence from Ethiopia and other settings was treated explicitly as contextual rather than Somalia-specific. Sources were considered according to their relevance to the review objectives, methodological credibility, contextual applicability, and contribution to understanding surveillance, preparedness, governance, financing, One Health coordination and equity. Preprints, duplicate publications, purely clinical treatment studies without surveillance or preparedness relevance, unsupported opinion pieces, and unrelated sources were excluded.

## Narrative synthesis and analytical approach

Evidence was synthesised narratively across disease pathways, existing early warning assets, the warning-to-action gap, governance and financing barriers, One Health and equity considerations, implementation priorities, and research needs. Interpretation considered differences in study design, setting, data source, exposure definition, disease outcome, and surveillance indicators. Formal risk-of-bias tools, duplicate independent screening, and quantitative pooling were not applied because the review was designed as a critical, policy-oriented narrative synthesis rather than as a systematic review. Analytical rigour was strengthened by distinguishing Somalia-specific findings from contextual evidence, explicitly identifying uncertainty and evidence gaps, and limiting conclusions to claims supported by the available literature.

## Climate-sensitive infectious disease risks in Somalia

The climate-sensitive infectious disease risk in Somalia is best understood through the interacting pathways that link rainfall anomalies, drought, flooding, temperature shifts, WASH disruption, displacement, food insecurity, vector ecology, livestock systems, and reduced access to healthcare. Climate hazards rarely act alone; they amplify transmission by reshaping exposure and weakening health system responses, especially in fragile and conflict-affected settings (1, 15–17). In Somalia, where recurring climate shocks intersect with large displaced populations and constrained public services, surveillance must track ecological and social precursors alongside case counts and not treat climate as a stand-alone cause of outbreaks.

Cholera, acute watery diarrhoea, and related diarrhoeal diseases are the most obvious climate-linked threats. Flooding can contaminate water sources and overwhelm sanitation systems, while drought pushes households and IDP communities toward unsafe water points and reduces hygiene capacity (16, 18). WHO EMRO situation reports have described continued cholera/AWD transmission following the 2023 outbreak wave, including spread into flood-affected districts and major risks linked to inadequate safe water and sanitation among displaced populations (19). For Somalia, the surveillance implications are straightforward: rainfall and flood alerts, WASH breakdown, displacement density, and diarrhoeal clusters should be monitored together so that water treatment, case management, and community response can be triggered earlier.

Malaria and dengue require different surveillance lenses because their risk pathways are mediated by rainfall, standing water, temperature, and vector ecology. The WHO's Somalia EPI Watch already links epidemic-prone disease monitoring with drought-affected districts, where malaria, acute diarrhoeal disease, and measles are tracked alongside community health worker alerts (20). Dengue has been confirmed in Puntland and Banadir, and outbreak investigations have identified stagnant water, containers, and other household breeding sites as important local risks (21, 22). Modelling from Ethiopia provides contextual evidence on the climatic suitability of dengue transmission, while broader African evidence suggests that climate change may shift vector-borne disease risk from malaria towards arboviruses; these findings should not be interpreted as Somalia-specific evidence (23, 24). Climate-informed surveillance here should combine climate data, fever syndromic reporting, entomological monitoring, diagnostic readiness, and district risk mapping.

Rift Valley fever is one of Somalia's most important zoonotic climate-sensitive risks and requires a One Health approach. In Somalia's 2023 zoonoses prioritisation, RVF was ranked first among seven priority zoonoses, reflecting the country's livestock dependence and the need for integrated human-animal-environment surveillance (25). Serological evidence of RVF in ruminants from Afgoye and Jowhar indicates exposure at the animal interface, although the human burden remains poorly characterised (26). Because the risk increases with heavy rainfall, flooding, livestock movement, and mosquito proliferation, surveillance should connect animal abortion events, livestock mortality, vector signals, and human febrile alerts before human cases appear (27, 28).

Measles, diphtheria, polio, and other vaccine-preventable diseases are not directly climate-driven; however, climate shocks can amplify their risk through displacement, malnutrition, overcrowding, and interruption of immunisation services. Somali studies show that IDP vaccination uptake is shaped by household, service, and policy barriers, while zero-dose children are concentrated in nomadic, remote, and conflict-affected populations (29–31). This distinction matters: directly climate-sensitive infections require environmental, vector, or WASH triggers, whereas indirectly climate-amplified vaccine-preventable diseases require displacement, nutrition, and immunisation triggers. Disease-specific risk pathways are only useful if Somalia's surveillance and early warning systems can convert them into timely preparedness actions. Table 1 summarises the disease-specific pathways, warning signals, preparedness actions, and supporting evidence for the main directly climate-sensitive and indirectly climate-amplified infectious disease groups discussed in this review.

**Table 1:** Climate-sensitive and indirectly climate-amplified infectious disease groups, warning signals, and preparedness actions in Somalia.

Disease group	Risk pathway in Somalia	Key warning signals	Early preparedness actions	References
Cholera/Acute Watery Diarrhoea Directly climate-sensitive	Flooding can contaminate water sources and disrupt sanitation; drought can push IDP and pastoral communities toward unsafe water points and reduce hygiene capacity; dense IDP settlements may amplify transmission.	Flood and drought alerts; worsening food insecurity and displacement; WASH breakdown reports in IDP settlements; diarrhoeal cluster notifications from EWARN or community reporters.	Activate water-quality surveillance and chlorination; pre-position oral rehydration supplies and cholera kits; intensify community health worker alerts in flood- or drought-affected districts; coordinate WASH Cluster response.	(12, 16, 20, 32)
Malaria Directly climate-sensitive	Seasonal rainfall, flooding, standing water, and temperature influence Anopheles breeding and malaria transmission, particularly where displacement and disrupted access to prevention and treatment increase vulnerability.	Above-average rainfall forecasts; flooding and standing-water accumulation; increases in suspected malaria or febrile illness; community alerts; changes in test positivity where available.	Intensify malaria surveillance and diagnostic readiness; pre-position rapid diagnostic tests and antimalarial supplies; strengthen vector-control preparedness; prioritise displaced and hard-to-reach populations.	(20, 24, 32)
Dengue Directly climate-sensitive	Rainfall, household water storage, stagnant containers, urban crowding, and temperature can increase *Aedes* breeding and dengue transmission; outbreaks have been documented in Banadir and Puntland.	Rainfall anomalies; household and peri-domestic breeding sites; febrile illness clusters; community alerts; entomological signals where available.	Strengthen febrile illness surveillance and dengue diagnostic readiness; conduct targeted *Aedes* source reduction; pre-position outbreak-investigation supplies; support laboratory confirmation and district risk mapping.	(21–24, 33)
Rift Valley Fever Directly climate-sensitive — One Health	Heavy rainfall and flooding can create Aedes and Culex mosquito breeding conditions; livestock movement and close human–animal contact amplify zoonotic spillover risk; serological evidence of RVF has been documented in ruminants from Afgoye and Jowhar.	Livestock abortion events; unexplained animal mortality in pastoral areas; heavy rainfall and flooding alerts; vector activity signals; community reports of febrile illness with haemorrhagic features.	Activate joint human–animal investigation teams; intensify livestock surveillance and serological monitoring; enhance mosquito vector surveillance; ensure laboratory diagnostic readiness for RVF confirmation.	(14, 25–28)
Measles, Diphtheria, and Polio Indirectly climate-amplified vaccine-preventable diseases	These diseases are not directly climate-driven; climate shocks may amplify risk through displacement, malnutrition, overcrowding in IDP settlements, cold-chain disruption, and reduced healthcare access; zero-dose children are concentrated among nomadic, remote, and conflict-affected populations.	IDP influx; IPC AMN Phase 3 + acute malnutrition classification; low immunisation coverage; cold-chain disruption; zero-dose cluster identification in IDP, nomadic, or hard-to-reach communities.	Activate mobile vaccination outreach in IDP settlements and remote areas; conduct zero-dose child mapping; strengthen cold-chain monitoring; deploy community health workers for case finding and symptom surveillance.	(12, 34–36)

This table summarises the main directly climate-sensitive and indirectly climate-amplified infectious disease groups discussed in the review. It links each disease group to risk pathways, early warning signals, and preparedness actions relevant to district-level, multisectoral risk assessment in Somalia. Climate signals should be interpreted alongside WASH, displacement, nutrition, livestock, laboratory, community, and health-system indicators, rather than as independent outbreak predictors. The table is intended to guide preparedness planning and does not establish definitive causal thresholds. AFI, acute food insecurity; AMN, acute malnutrition; AWD, acute watery diarrhoea; EWARN, Early Warning Alert and Response Network; IDP, internally displaced person; IDSRS, Integrated Disease Surveillance and Response System; IPC, Integrated Food Security Phase Classification; RVF, Rift Valley fever; WASH, water, sanitation, and hygiene.

## Existing surveillance and early warning systems

Climate-informed surveillance in Somalia should build on, rather than replace, the existing architecture. Across disease reporting, climate monitoring, WASH assessment, food security intelligence, displacement tracking, nutrition surveillance, and livestock signals, Somalia has meaningful building blocks. The core challenge is integration: these systems operate in largely separate institutional channels, and upstream risk signals do not consistently trigger coordinated district-level preparedness before outbreak thresholds are crossed (12, 37). The health surveillance core rests on the EWARN and IDSRS, each representing a layer of capacity built incrementally since 2008 and 2016, respectively. A formal evaluation of Somalia's EWARN from 2017 to 2020 documented meaningful progress: the proportion of timely reports rose from 24.6% in 2017 to 96.8% by 2020, and alert verification improved from 2.0% to 52.6% over the same period (32). The DHIS2 has been configured to support aggregate weekly disease reporting at the district level, and the IDSRS review identified coordination mechanisms and standardised tools as key implementation gains between 2016 and 2023 (12). However, the data completeness of the EWARN averaged below 60% across all four evaluation years, and the system remains insufficiently integrated with non-health warning platforms, a structural gap that constrains its value for climate-informed preparedness (32). The WHO and Health Cluster weekly epidemiological bulletins provide supplementary epidemic intelligence for alert-prone conditions, including AWD/cholera, measles, malaria, dengue, diphtheria, and polio, although continued reliance on humanitarian and partner-supported reporting underscores the need for stronger institutional integration (20).

In Somalia's insecure, displacement-affected, and hard-to-reach areas, community-based and humanitarian surveillance is often the first source of actionable signals. Evidence from comparable conflict settings shows that community-based surveillance can generate timely alerts and capture displacement, malnutrition, and other community events that routine facility reporting may miss (38, 39). Systematic reviews confirm that community health workers in fragile and humanitarian settings contribute to outbreak detection, case finding, and surveillance continuity in populations otherwise invisible to routine systems (40, 41). In Somalia, these networks, including IDP focal points, local NGO staff, and frontline facility workers, are foundational assets for any climate-informed system.

Upstream risk signals from outside the health sector provide essential early warning. FEWS NET provides an established drought early warning capacity (42), whereas FEWS NET and FSNAU together generate regularly updated food security, climate risk, and displacement intelligence for Somalia. An ecological study of IDP out-migration in Somalia found strong associations between failed rains and population movement at a three-month lag and food insecurity at a one-month lag, temporal patterns with direct implications for outbreak preparedness planning (43). WASH cluster monitoring, One Health zoonotic prioritisation, and livestock surveillance generate additional signals relevant to RVF and other climate-linked diseases (25, 26). None of these streams are routinely fused with disease surveillance at the district level or with each other.

Therefore, Somalia's surveillance challenge is not the absence of warning systems but rather their fragmented institutional connectivity. Somalia's One Health national bridging workshop identified joint surveillance systems and emergency funding structures as priority unmet needs (37). The result is a warning-to-action gap: climate, WASH, displacement, nutrition, and livestock signals exist but do not reliably convert into funded, district-level, disease-specific preparedness, which is the core policy and implementation failure examined in the following section.

## From warning to action: the core implementation gap

As established in the preceding section, Somalia is not without early warning capacity. Disease case reports, climate forecasts, food security classifications, displacement data, nutrition surveillance, livestock signals, and community alerts are generated, often in parallel and with varying degrees of timeliness. The critical gap is institutional, not informational: these signals do not reliably translate into funded, district-level, disease-specific preparedness actions before transmission thresholds are crossed. A global analysis of 64 infectious disease warning and response systems found that 80% were reactive, designed to respond after case counts rise, and only 6% were prevention-centred, linking upstream risk signals to strategies that addressed the drivers of disease emergence (44). Somalia's challenges are a sharp illustration of this broader pattern.

The disease-specific warning signals and corresponding preparedness actions are summarised in Table 1. Across these disease groups, the central problem is not the absence of relevant signals but the lack of institutional mandates, pre-agreed authority, and financing to activate preparedness before case counts rise.

Converting warnings into action requires specific governance infrastructure: pre-agreed, disease-specific risk thresholds; designated lead institutions responsible for triggering each response; pre-positioned supplies and trained teams; and flexible pre-allocated financing that can be disbursed without requiring post-event budget approvals. The anticipatory action literature consistently demonstrates that trigger reliability, flexible funding, and local institutional ownership are key enabling conditions for converting early warnings into early action, and that their absence produces predictable and preventable delays (45–47). OCHA's anticipatory action pilots, which explicitly include Somalia, confirm these lessons, while also noting that forecast resolution and lead times remain limiting factors at the subnational scale (45). However, these enabling conditions have not yet been reliably institutionalised in Somalia. Currently, no consistently institutionalised district-level mechanism synthesises climate, WASH, displacement, nutrition, and livestock signals into shared outbreak risk assessments (12, 37). Displacement-affected, pastoralist, and hard-to-reach populations — precisely those most exposed to compound climate and disease shocks — remain disproportionately outside both the surveillance network and preparedness planning frameworks. These structural deficits point to a set of policy, governance, and financing barriers, which are examined in the following section.

## Policy and operational barriers to climate-informed surveillance

Somalia's warning-to-action gap cannot be resolved through surveillance technology alone. This reflects a governance architecture that is not yet fully configured for integrated climate-informed risk assessment. Disease surveillance, food security monitoring, WASH assessment, displacement tracking, nutrition surveillance, livestock health reporting, hydrometeorological forecasting, and disaster management each operate through separate mandates, funding lines, and reporting hierarchies, with no consistently institutionalised routine mechanism for synthesising these streams into a shared district-level outbreak risk picture. Somalia's 2023 IHR–PVS National Bridging Workshop identified joint surveillance systems for selected One Health threats, high-level ministerial coordination, and emergency funding structures as priority objectives, underscoring the institutional gaps that continue to limit integrated warning-to-action capacity (37). The broader One Health governance literature recognises that sectoral and institutional silos, compounded by fragmented financing and chronic underinvestment in prevention, are the primary structural barriers to integrated threat management (48). At the district level, where preparedness actions must ultimately be implemented, operational capacity remains constrained. Many district health teams lack adequately trained personnel, diagnostic equipment, reliable connectivity, and logistical resources for timely alert verification and rapid response. Insecurity restricts physical access to conflict-affected and remote areas, creating systematic gaps in case ascertainment and response (35). The laboratory capacity for confirming climate-linked pathogens remains concentrated in a small number of facilities, and sample referral delays extend the interval between initial signal detection and confirmed diagnosis (14). Evidence from comparable conflict-affected settings indicates that early warning systems that lack disease-specific, locally calibrated alert thresholds have limited operational utility in triggering timely preparedness actions (49).

Financing is a major structural constraint. Somalia's IDSRS implementation progress stands at only 15% for the financing domain — the lowest across all assessed dimensions — reflecting prolonged over-reliance on short-term, project-cycle humanitarian funding (12). This financing structure impedes sustained investment in surveillance staffing, supply pre-positioning, routine supervision, and flexible pre-allocated budgets required for anticipatory preparedness. Without domestically owned emergency funding structures, response activities remain contingent on external and often unpredictable donor cycles rather than on institutionally agreed risk thresholds.

The institutionalisation of One Health across human health, animal health, the environment, and meteorology remains nascent. Somalia's One Health initiatives continue to depend heavily on external donor support and have not been fully embedded within government planning or budgeting systems at the federal, state, or district levels (14, 50). For Rift Valley fever and other priority zoonoses, animal health and environmental signals that could serve as early human health warnings are not routinely shared with the health sector or integrated into district epidemic risk assessments (25, 37). A community-based One Health surveillance model piloted in Adadle district, Ethiopia, demonstrated that joint human–animal–community surveillance is operationally feasible at the district level when sectors are co-trained and structurally linked; however, replication across Somalia would require sustained political commitment and domestic financing that remains insufficiently institutionalised (51).

These barriers fall most heavily on populations bearing the greatest climate-sensitive disease risks. Internally displaced persons, nomadic and agropastoral communities, malnourished children, and populations in insecure or hard-to-reach areas are often less visible in routine surveillance systems and less likely to be reached by early response measures (34, 35). Zero-dose and under-vaccinated children concentrated in IDP settlements remain acutely vulnerable when climate shocks compound immunisation disruption, yet mobile vaccination and community outreach remain inconsistently activated in these areas. The movement patterns of pastoralist communities and their close human-animal-environment interface represent an underutilised early warning resource that formal surveillance systems rarely incorporate (52, 53). Addressing these equity dimensions is not merely an ethical priority; it is a functional requirement for any climate-informed surveillance system capable of detecting risk in areas where climate-sensitive disease risk is highest. The preparedness priorities proposed in the following section are directly informed by these governance, operational, financing, and equity gaps.

## Implementation priorities for Somalia

Based on the evidence reviewed, implementation should prioritise four enabling conditions: integrated district risk assessment, strengthened district and community surveillance capacity, institutionalised One Health coordination with anticipatory financing, and equity-centred outreach to the most vulnerable populations. These priorities should build on Somalia's existing EWARN and IDSRS/DHIS2 architecture rather than create parallel surveillance systems (12, 32). Disease-specific warning signals and early preparedness actions are summarised in Table 1.

District surveillance capacity and early community warning are essential. Trained district surveillance officers with clear risk-assessment mandates, rapid response team readiness, functional sample transport, and digital reporting adapted to low-connectivity environments are the minimum requirements. Community health workers, IDP settlement focal points, and frontline facility staff should act as formal signal reporters. Evidence from the Adadle district, Ethiopia, indicates that co-training community animal and human health workers within a joint One Health surveillance unit can strengthen the district-level detection of zoonotic diseases (51). Nomadic and pastoralist communities, a documented blind spot in formal surveillance systems, should be systematically incorporated through mobile outreach and community informant networks (52).

One Health coordination and anticipatory financing must be institutionalised. Somalia's 2023 One Health roadmap identified joint surveillance systems and emergency funding structures as its highest-priority objectives (37). Routine joint risk assessment across the health, livestock, environment, WASH, food security, and disaster management sectors should directly inform district preparedness. Pre-positioned commodities and trigger-based financing mechanisms — drawing on broader lessons from OCHA-supported anticipatory action frameworks, including pilots in Somalia — can enable preparedness action before outbreak thresholds are crossed rather than after (45, 46).

Underpinning these priorities is the imperative of equity in health. Somalia's internally displaced persons, nomadic and pastoralist communities, malnourished and zero-dose children, and populations in insecure or hard-to-reach areas face high climate-sensitive disease risk and weak surveillance coverage. District risk assessments must disaggregate vulnerability by population subgroup; mobile vaccination and community outreach should be activated through pre-agreed triggers; and community-based surveillance must be explicitly designed to reach populations that routine systems often miss (35, 53). Together, these implementation priorities provide the enabling conditions for the climate-informed surveillance-to-action framework described below.

## Proposed climate-informed surveillance-to-action framework

The proposed framework does not require the establishment of new institutions to implement it. It builds on Somalia's existing surveillance assets: the EWARN network, IDSRS/DHIS2, community-based reporting structures, FEWS NET and FSNAU food security and nutrition intelligence, WASH monitoring, displacement tracking, livestock health reporting, and the emerging One Health coordination architecture (12, 14, 32).

Its defining feature is functional integration: these currently fragmented data streams are linked at the district level into a shared risk assessment platform, and the output of that assessment is not a report but a decision — whether or not to activate a pre-agreed preparedness response. The framework's central departure from current practice is reframing surveillance as an anticipatory, decision-oriented function rather than a passive data collection system (54). The framework operates in five linked stages. First, **multisource signal detection** combines climate forecasts, hydrometeorological alerts, WASH conditions, displacement movements, nutrition and food security intelligence, livestock morbidity and mortality reports, laboratory information, and community-based event signals. Second, **integrated district risk assessment** interprets these signals jointly with EWARN/IDSRS surveillance data and assesses their convergence within the local epidemiological and vulnerability context. Third, a **disease-specific trigger decision** determines whether the combined evidence meets pre-agreed criteria for preparedness action. Fourth, **early preparedness** action activates the relevant response package, including commodity pre-positioning, rapid-response team mobilisation, laboratory or sample-referral readiness, and targeted community outreach. Fifth, **feedback, accountability, and learning** assess whether actions occurred on time and use implementation experience to refine trigger criteria and the signal mix (47, 55).

An illustrative composite trigger for a flood-prone district along the Jubba or Shabelle rivers could be activated when an official flood warning coincides with documented deterioration in safe water or sanitation conditions and an increase in community or EWARN acute watery diarrhoea alerts (16, 18, 19, 32). This would constitute the technical trigger for action; the exact rainfall, river level, WASH, and surveillance thresholds would require local calibration and prospective validation. The corresponding governance trigger should specify the responsible authority, financing mechanism, preparedness package, and indicators for timely implementation, consistent with anticipatory action and action-oriented early warning principles (45, 46, 55). This example is an operational template rather than a validated, national threshold.

**Figure 1:**
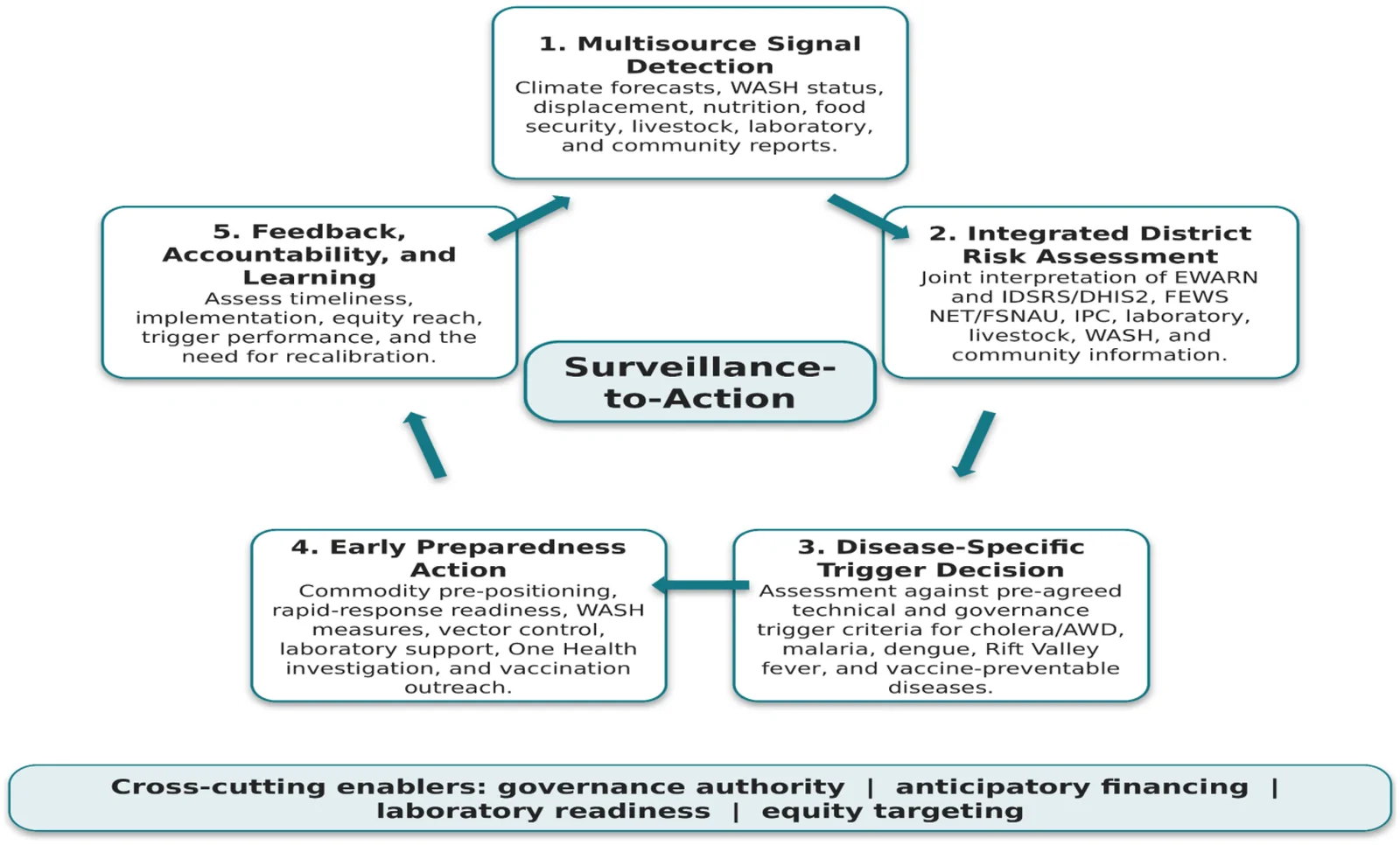
Climate-informed surveillance-to-action framework for Somalia. The framework links multisource signal detection to integrated district risk assessment, disease-specific trigger decision, early preparedness action, and feedback, accountability, and learning. It emphasises that surveillance should not end with reporting, but should support timely, accountable action before outbreaks escalate. Equity-focused implementation should prioritise internally displaced persons, pastoralist and nomadic communities, zero-dose children, malnourished populations, and hard-to-reach areas.

The proposed surveillance-to-action framework is summarised in Figure 1.

The framework's operational viability depends on governance and accountability conditions that are currently underdeveloped in Somalia. Institutional ownership must be unambiguous: the framework should be formally anchored within the National Action Plan for Health Security, with the Ministry of Health holding the coordination authority. District surveillance teams should have clear authority within the agreed protocols to activate preparedness responses without waiting for national confirmation. Routine joint risk-assessment meetings convening health, livestock, environment, WASH, food security, and humanitarian actors must be embedded in the district operational calendar rather than convened only in response to emerging crises.

This framework reframes climate-informed surveillance in Somalia as an anticipatory public health function that links environmental and population signals to pre-agreed trigger criteria, shared risk assessments, and accountable, early action. Rather than replacing existing systems, it requires that they be connected, interpreted jointly, and used to act promptly. The Discussion section examines the feasibility constraints, transferability to comparable fragile settings, and broader implications of this model for climate-resilient health system design in conflict-affected and climate-vulnerable contexts.

## Discussion: lessons for fragile and climate-vulnerable health systems

Somalia's recurrent infectious disease outbreaks reflect not only climate hazards but also the convergence of drought, flooding, and rainfall anomalies with WASH insecurity, acute malnutrition, mass displacement, immunisation disruption, and fragile surveillance systems (32, 35). The central contribution of this review is a shift in the analytical frame: the key challenge is not that warning signals are absent — EWARN, FEWS NET, IPC, WASH monitoring, and livestock surveillance all generate relevant intelligence — but that Somalia lacks consistently institutionalised mechanisms to convert those signals into timely, disease-specific preparedness actions. Somalia's experience illustrates this warning-to-action gap in a fragile and climate-vulnerable context.

The implications for Somalia's health security planning are significant. Anchoring a climate-informed surveillance-to-action model within the National Action Plan for Health Security would institutionalise multisectoral risk assessment as a routine government function. The IDSRS/DHIS2 platform provides a scalable foundation; however, financing remains a major implementation constraint (12). Sustainable investment in district surveillance capacity, pre-positioned commodities, and pre-agreed preparedness triggers is essential for any anticipatory model. Financing should combine progressive domestic ownership with predictable pooled, external, and anticipatory financing rather than depend on domestic resources alone (45). One Health governance, currently dependent on external funding and weakly institutionalised across government sectors, must be embedded in district structures and primary healthcare systems rather than sustained as a parallel project architecture (14, 50).

Somalia's case offers transferable lessons for other fragile and climate-vulnerable nations. Climate-informed surveillance must be realistic, low-burden, district-oriented, community-linked, and integrated into existing systems. The Syria EWARN experience confirms that disease-specific alert thresholds can improve sensitivity but require deliberate local calibration and sustained institutional commitment (49). Global pandemic preparedness frameworks that do not explicitly incorporate fragile and conflict-affected settings risk failing populations that are most exposed to climate-amplified infectious disease threats (56). Nomadic and pastoralist communities often remain less visible in formal surveillance architectures across Africa (52, 53), and Somalia is no exception: equity-driven surveillance design is an epidemiological necessity, not an aspirational goal.

Implementation of this framework also carries practical risks. Incomplete or insufficiently granular data, limited subnational forecast resolution, and poorly calibrated triggers may result in inappropriate activation or missed actions (45, 55). These uncertainties support phased implementation, local validation of disease-specific trigger criteria, routine performance review, and periodic recalibration, rather than immediate nationwide adoption of fixed thresholds (45, 49, 55).

Several important research gaps remain. This review identified no validated Somalia-specific climate–disease threshold models for cholera/AWD, malaria, dengue, or Rift Valley fever. Evidence on the effectiveness of community-based early warning systems in IDP settlements and pastoralist contexts remains limited. Evidence on integrating the IDSRS/DHIS2 with climate, WASH, displacement, nutrition, and livestock data is limited. Cost-effectiveness evidence on anticipatory preparedness vs. reactive response remains limited for the Somali setting, as are documented governance and financing models for institutionalised warning-to-action systems in conflict-affected contexts.

This review has some limitations. The evidence base is heterogeneous and relies substantially on grey literature and official surveillance documents, and predictive studies specific to Somalia are sparse. Underreporting from insecure and hard-to-reach areas likely distorts both burden estimates and surveillance performance assessments. Isolating the effects of climate change from those of conflict, displacement, poverty, and health system weaknesses remains methodologically difficult. These constraints reinforce, rather than undermine, the review's central argument: the value of climate-informed surveillance in Somalia should be judged by whether it reliably converts early warnings into equitable and accountable public health actions.

## Conclusion

Somalia's climate-sensitive infectious disease burden emerges from the convergence of climate shocks with WASH insecurity, acute malnutrition, mass displacement, conflict, interrupted immunisation and fragile health services, not from climate alone. Cholera, malaria, dengue, and Rift Valley fever are substantially modulated by rainfall, flooding, drought, and vector ecology. Measles, diphtheria, and polio are amplified when climate shocks compound displacement, malnutrition, and immunisation breakdown. Managing these intersecting risks requires more than improved case detection; it requires an anticipatory surveillance function that integrates climate forecasts, WASH monitoring, displacement and nutrition data, livestock signals, laboratory capacity, and community reporting into earlier, district-level, and disease-specific preparedness actions.

Somalia's existing surveillance architecture — EWARN, IDSRS/DHIS2, FEWS NET and FSNAU intelligence, One Health platforms, and community-based reporting structures — provides a foundation. The critical deficit is not the absence of signals but the failure to convert them into timely, funded, and coordinated actions before transmission thresholds are crossed. Closing this warning-to-action gap requires pre-agreed disease-specific preparedness triggers, institutionalised joint risk assessment across health, livestock, WASH, food security, and humanitarian actors, flexible pre-financed response mechanisms, and equity-centred design that explicitly reaches internally displaced persons, nomadic and pastoralist communities, zero-dose and malnourished children, and populations in hard-to-reach and insecure areas. Somalia's 2023 One Health national roadmap and the IDSRS/DHIS2 platform provide important policy entry points for this model. What remains is institutional commitment and sustainable financing arrangements that combine progressive domestic ownership with predictable external and anticipatory support. The goal of climate-informed surveillance in Somalia is not to detect outbreaks faster after climate shocks have already converged with humanitarian vulnerability; rather, it is to act before this convergence becomes an emergency.
